# Pentavalent Single-Domain Antibodies Reduce *Campylobacter jejuni* Motility and Colonization in Chickens

**DOI:** 10.1371/journal.pone.0083928

**Published:** 2013-12-31

**Authors:** Ali Riazi, Philippa C. R. Strong, Russell Coleman, Wangxue Chen, Tomoko Hirama, Henk van Faassen, Matthew Henry, Susan M. Logan, Christine M. Szymanski, Roger MacKenzie, Mehdi Arbabi Ghahroudi

**Affiliations:** 1 AbCelex Technologies Inc., Toronto, Ontario, Canada; 2 Human Health Therapeutics, National Research Council of Canada, Ottawa, Ontario, Canada; 3 Dow AgroSciences, Indianapolis, Indiana, United States of America; 4 Centennial Centre for Interdisciplinary Science, Department of Biological Sciences and Alberta Glycomics Centre, The University of Alberta, Edmonton, Alberta, Canada; 5 School of Environmental Sciences, University of Guelph, Guelph, Ontario, Canada; 6 Department of Biology, Carleton University, Ottawa, Ontario, Canada; Wadsworth Center, New York State Dept. Health, United States of America

## Abstract

*Campylobacter jejuni* is the leading cause of bacterial foodborne illness in the world, with symptoms ranging from acute diarrhea to severe neurological disorders. Contaminated poultry meat is a major source of *C. jejuni* infection, and therefore, strategies to reduce this organism in poultry, are expected to reduce the incidence of *Campylobacter*-associated diseases. We have investigated whether oral administration of *C. jejuni*-specific single-domain antibodies would reduce bacterial colonization levels in chickens. Llama single-domain antibodies specific for *C. jejuni* were isolated from a phage display library generated from the heavy chain IgG variable domain repertoire of a llama immunized with *C. jejuni* flagella. Two flagella-specific single-domain antibodies were pentamerized to yield high avidity antibodies capable of multivalent binding to the target antigen. When administered orally to *C. jejuni*-infected two-day old chicks, the pentabodies significantly reduced *C. jejuni* colonization in the ceca. *In vitro*, the motility of the bacteria was also reduced in the presence of the flagella-specific pentabodies, suggesting the mechanism of action is through either direct interference with flagellar motility or antibody-mediated aggregation. Fluorescent microscopy and Western blot analyses revealed specific binding of the anti-flagella pentabodies to the *C. jejuni* flagellin.

## Introduction

*Campylobacter jejuni*, a Gram negative bacterium, is currently one of the most prevalent foodborne pathogens and the leading cause of bacterial gastroenteritis in humans worldwide [1]. In North America, campylobacteriosis outnumbers the reported cases of illnesses caused by *Salmonella, Shigella, Listeria* and *Escherichia coli* combined [2]–[4]. Despite relatively mild diarrheal illness, *C. jejuni* infection has been associated with severe long-term complications, including: Guillain-Barré Syndrome [5]–[7], reactive arthritis and inflammatory bowel disease [8], [9]. It is estimated that between 50–80% of human campylobacteriosis cases can be attributed to consumption of contaminated chicken, and therefore meat from broiler chickens is considered the primary vector for transmitting the pathogen to humans [10]–[12].

Reduction of *C. jejuni* levels in poultry decreases the incidence of *Campylobacter*-associated gastroenteritis [12], [13]. Various intervention strategies targeting different stages of poultry meat production are currently under investigation. To date the most accepted strategies work by preventing *Campylobacter* spp. from entering the flock through installation of hygiene barriers and fly screens, use of high quality water, reduction of slaughter age, and discontinuation of thinning practices [12], [14]–[17]. However, the susceptibility of chickens to infection by *C. jejuni* and its ubiquity in the environment have negatively impacted the success of biosecurity-based approaches, highlighting the need for alternative approaches by which the bacterial infection can be controlled or eliminated [3], [18], [19]. Antibiotics such as fluoroquinolones and macrolides have been approved for the control of *Campylobacter* spp. in both poultry and humans. However, their prolonged use in humans and animals has led to a rapid increase of resistant strains in many countries around the world and their use is no longer recommended in animal feed stocks [20]–[22]. Application of *Campylobacter*-specific lytic bacteriophages has been proposed as an alternative strategy. A reduction in cecal *C. jejuni* levels of 0.5–5 log_10_ CFU/g has been reported when bacteriophages were administered to chickens as feed-additives or veterinary drugs [23]–[25]. Development of resistance, however, is considered to be a potential drawback of phage therapy and has been reported following phage treatment in several studies [26], [27]. In addition, finding a phage cocktail that would kill all *C. jejuni* strains is unlikely. Bacteriocins, which are proteinaceous substances produced by bacteria that inhibit growth, have also been extensively studied. Addition of bacteriocins to poultry drinking water completely eliminated the pathogen in 90% of cases or reduced its levels by 10^6^-fold or more [28]. Other biological reagents such as probiotics [29], [30] and plant bioactive compounds [31], [32] have also been used as food or water additives and have been shown to reduce *Campylobacter* loads in chickens. The bactericidal effects of probiotic strains such as lactic acid bacteria against *C. jejuni* have been attributed to the production of organic acids and bacteriocins or bacteriocin-like substances [29], [33]. Medium chain fatty acids such as caprylic acid and monoacylglycerols are alternatives to antibiotics that have been used as feed and water additives to control or eliminate *Campylobacter* loads in chickens [34]–[36]. However, despite the reported efficacies none of these compounds have been widely adopted in the field due to inconsistency or lack of data on efficacy, safety, toxicity, scale-up production and purification, and the development of resistance [12] (reviewed in [14]). Furthermore, other methods of intervention such as using vaccines [37]–[41], competitive exclusion [12], [14], [37], [42], [43] or producing genetically engineered *Campylobacter*-resistant chickens [44] have had limited success in preventing *C. jejuni* colonization in chickens, and therefore, have not been commercialized.

Antibodies were originally recognized as effective antimicrobial reagents by Behring and Kitasato in the early 1890s [45], [46] and since then, serum therapy became an effective strategy to combat many infectious diseases. The presence of specific antibodies in the serum or intestinal secretions has been associated with resistance of rabbits [47]–[49] and mice [49], [50] to colonization by *C. jejuni*. In young chickens (less than 2–3 weeks of age), the presence of maternal antibodies against *Campylobacter* delays the onset of colonization and reduces the rate of horizontal spread of *C. jejuni* in the flock [19], suggesting that passive immunotherapy using anti-*Campylobacter* antibodies could be an attractive approach for interfering with bacterial colonization in chickens. Indeed, passive immunization with anti-flagella monoclonal antibodies has already been shown to reduce *C. jejuni* colonization in mice [51]. Similarly, the use of hyperimmunized anti-*C. jejuni* rabbit serum or anti-*C. jejuni* antibodies appears to be effective in diminishing the colonization in chickens [52]. Consistent with this, others have shown that poultry abattoir workers who have high titres of *Campylobacter*-specific IgGs circulating in their blood rarely become ill due to *Campylobacter* infection [53]. Despite all these facts, antibodies as preventive or therapeutic reagents for *Campylobacter* treatment and control have not gained market attention largely due to the high cost of manufacturing, sensitivity of conventional antibodies to gastrointestinal (GI) tract proteases, lack of effective GI tract delivery systems, and relatively high antigenic variation among *C. jejuni*, which requires multiple antibody preparations to target different strains.

For applications such as bacterial toxin neutralization and/or inactivation of infectious agents, antibody fragments (e.g., Fabs, scFvs, single-domain antibodies) are preferable to whole antibodies (e.g., IgGs) due to lower production cost in a bacterial system and ease of genetic manipulation. The smallest, naturally occurring antigen binding fragments are the variable domains of heavy chain antibodies found in camelids [54] and the immunoglobulin new antigen receptors in sharks [55], [56]. The antigen binding sites of these antibodies reside in a single domain. Camelid two chain antibodies, termed heavy chain only antibodies, have been extensively studied and their variable domains, termed V_H_Hs, also known as single domain antibodies (sdAbs) or nanobodies, have been shown to be extremely stable when cloned and expressed as monomers using recombinant expression systems [57], [58]. We have also previously shown that pentavalency can be conferred upon V_H_Hs by fusion of the V_H_H to a protein domain derived from the verotoxin B homopentamer [59]. The resulting pentabodies are compact, stable antigen-binding molecules and have high functional affinity (avidity). Additionally, the pentavalent antibodies are capable of enhancing agglutination when bound to antigens [59].

In the present study, we describe the isolation of V_H_H single domain antibodies specific for *C. jejuni* flagella from a phage-display antibody library. The pentameric forms of V_H_Hs were produced and characterized using various *in vitro* and functional assays. As well, the efficacies of orally administering these pentabodies in reducing *C. jejuni* colonization levels in chickens were evaluated.

## Results

### High Affinity V_H_H Antibodies Produced Against *Campylobacter* Antigens

ELISA analysis of the binding of the ELISA analysis of the binding of immune serum fractions, obtained by protein G and A chromatography of serum from day 95 after the immunization start, to *C. jejuni* flagella coated on microtitre plates showed that there were strong immune responses in the heavy chain antibody as well as the conventional antibody fractions when compared to the pre-immune serum collected before immunization on day 1 (data not shown).

**Figure 1:**
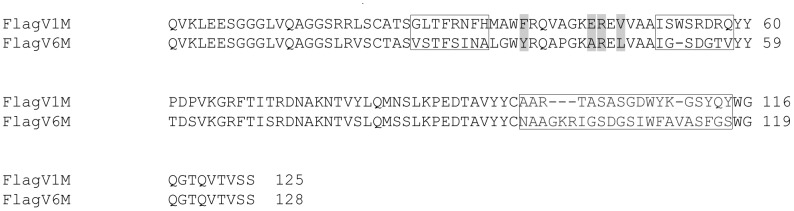
Sequence alignment of flagella-specific V_H_Hs. Amino acid sequence alignment of flagella–specific V_H_Hs isolated in this study. Framework regions (FRs) and complementarity determining regions (CDRs) (boxed) are aligned according to IMGT numbering system. Hallmark positions 42, 49, 50, and 52, distinguishing V_H_Hs from V_H_s, are highlighted in gray. The sequence alignment shows 65% protein sequence identity between the two V_H_Hs.

A V_H_H library with a size of 5×10^7^ individual transformants was constructed and analyzed for its complexity by sequencing 40 randomly selected colonies. All clones had inserts of expected sizes and were different from each other in their complementarity determining regions (CDRs). After four rounds of panning, individual colonies were screened by phage ELISA against flagella and V_H_H candidate clones were sequenced. Two unique flagella-specific sequences (FlagV1, FlagV6) were identified (GenBank™ accession numbers KF812523- KF812524), and all were determined to be V_H_Hs based on the presence of the *Camelidae* hallmark amino acids at positions 42 (F/Y), 49(E/Q/A), 50(R), 52(F/V/G/L) (IMGT numbering) (http://imgt.cines.fr) (Figure 1). The V_H_H binders were sub-cloned into expression plasmid vectors to allow targeted expression in the periplasm of *E. coli* TG1. Monomeric and pentameric V_H_Hs were produced at a yield of 10 to 80 mg/L of bacterial culture.

### Binding of V_H_H Antibodies to Flagella

**Figure 2:**
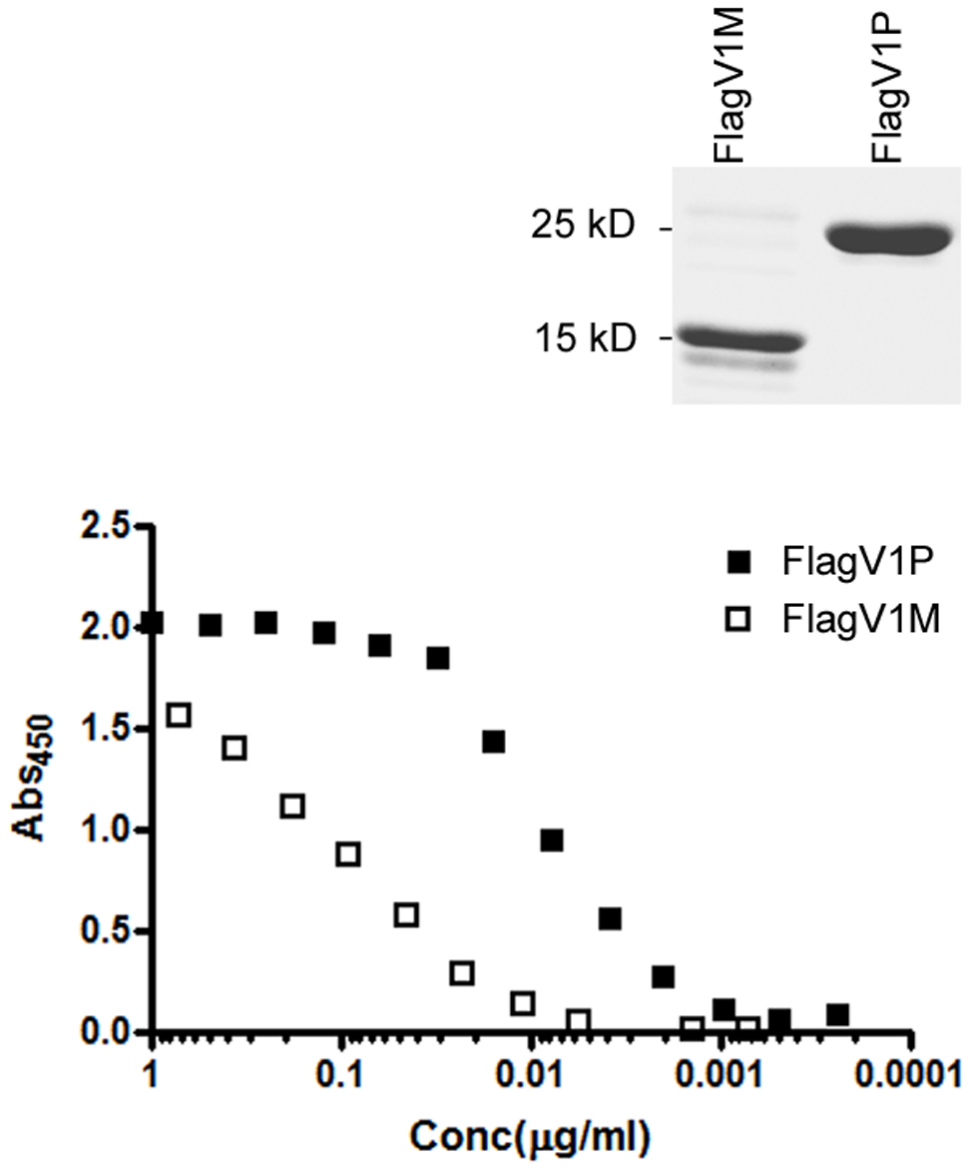
ELISA analysis of binding of the monomeric and pentameric V_H_Hs to *C. jejuni* surface antigens. Various concentrations (ranging from 10 to 0.0001 µg/ml) of the monomeric (FlagV1M) and pentameric (FlagV1P) forms of a flagella-specific V_H_H were used in ELISA. Absorbance data were normalized to represent equal amounts of the monomeric and pentameric V_H_H formats. Solid squares represent pentabody and open squares monomeric V_H_H. A representative image of Coomassie stained purified monomer and pentamer V_H_H is shown above the ELISA graphs.

**Figure 3:**
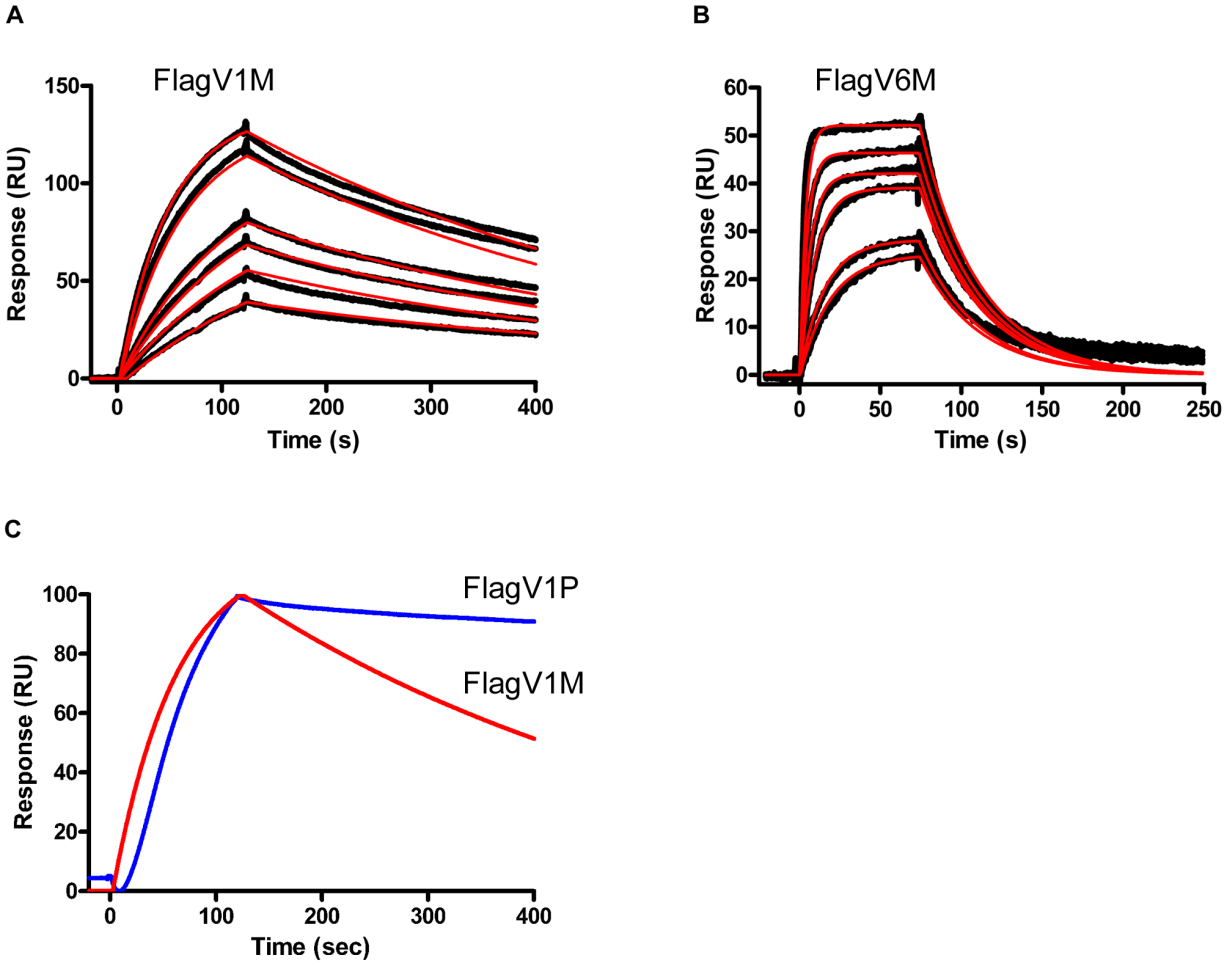
SPR analysis of the binding of the monomeric and pentameric V_H_Hs to antigen. Sensorgram overlays (A and B) represent the binding of 28, 42, 56, 70, 140 and 190 nM of FlagV1M and FlagV6M, respectively, to 700 RUs of biotinylated flagellin captured on an SA sensorchip. Black dots are the data points and the red curves are the fitting of the data to a 1∶1 interaction model. (C) The sensograms of FlagV1M (in red) and FlagV1P (in blue) were overlaid to show that a slower off-rate is obtained as a result of pentamerization indicating an increase in the functional affinity (avidity) of FlagV1P.

When expressed as monomers (M), two of the flagella binders (Flag V1M and FlagV6M) showed strong binding activity to flagella protein as determined by ELISA. Pentamerization (P) of FlagV1 V_H_Hs resulted in a further increase in their binding capacity as revealed by ELISA and surface plasmon resonance (SPR) (Figures 2 and 3). As shown, 50% maximum binding was achieved at 0.2 µg/ml (15.6 nM) FlagV1M and 0.005 µg/ml (40 pM) FlagV1P indicating an increase of almost 400-fold in functional affinity. The approximate affinity values for the monomeric V_H_Hs closely matches the values which were obtained by surface plasmon resonance (SPR) and shows that FlagV1M has an affinity in the range of 20–30 nM. Similar patterns of binding were observed in ELISA with monomeric and pentameric FlagV6 antibodies (data not shown). The bindings of V_H_Hs to biotinylated antigens captured on streptavidin surfaces were collected and analysed by SPR. The kinetic data for the V_H_Hs is presented in Table 1 and Figure 2.

**Table 1:** Rate and affinity constants for interaction of monomeric V_H_Hs with their respective antigens.

V_H_H	k_on_ (M^−1^s^−1^)	k_off_ (s^−1^)	K_D_ (M)
FlagV1M	1×10^5^	2×10^−3^	2×10^−8^
FlagV6M	1×10^6^	3×10^−2^	2.5×10^−8^

### Flagella-Specific V_H_Hs Recognize Non-Overlapping Epitopes

**Figure 4:**
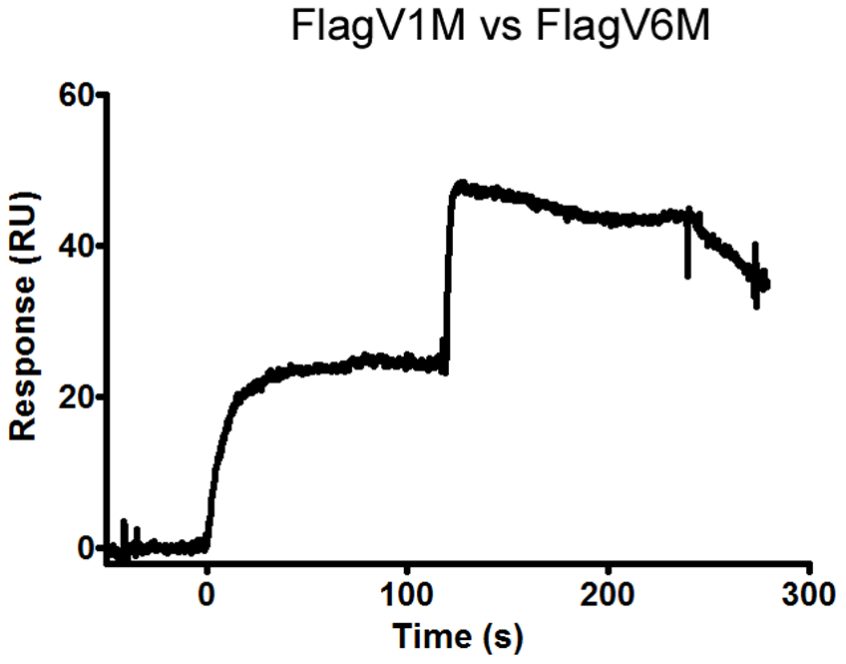
Epitope mapping of anti-flagella antibodies by simultaneous binding of flagella-specific V_H_Hs to the target antigen in SPR co-injection experiments. For the antigen, 60–100 µl of each V_H_H, at a concentration 50× its K_D_ value, were injected over 600–700 RUs of immobilized flagella at 30 µl/min. FlagV1 and FlagV6 appeared to bind distinct, non-overlapping epitopes since the signal approximately doubled with the second injection.

SPR analyses in which FlagV1M and FlagV6M V_H_H pairs were co-injected on a biotinylated flagella surface showed that FlagV6M is free to bind to flagella when FlagV1M is bound at saturating concentration as evidenced by almost doubling of the *R*_max_ indicating FlagV1M and FlagV6M recognize different epitopes (Figure 4).

### Fluorescence Microscopy Reveals Binding of FlagV1P and FlagV6P to the Assembled Flagella Filament

**Figure 5:**
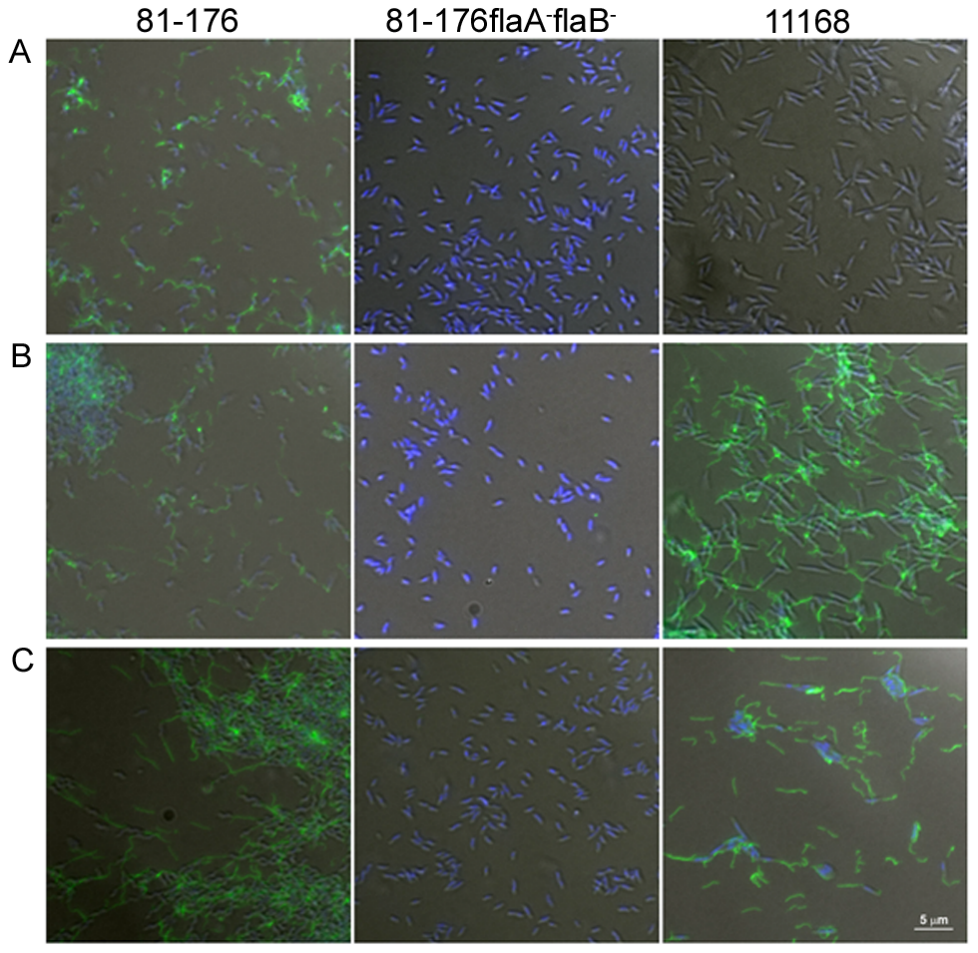
Binding of anti-flagella pentabodies to intact flagellar filament. Fluorescence microscopy showing FlagV1P (A) and FlagV6P (B) pentabody binding to *C. jejuni* flagella. Fluorescently labelled FlagV1P and FlagV6P were hybridized with either *C. jejuni* strain 81–176, 81–176 *flaA^−^flaB^−^*, or *C. jejuni* strain 11168. (C) Fluorescence microscopy showing polyclonal anti-81–176 flagella binding to both strains, 81–176 and 11168. Representative fields of view are shown for all images at the same magnification, as indicated by the 5 µm bar.

Fluorescently labelled FlagV1P bound flagella of *C. jejuni* strain 81–176, but no binding was observed with a *flaA^−^flaB^−^* mutant strain of 81–176 (Figure 5A). A similar pattern of binding was noted with FlagV6P (Figure 5B). Interestingly FlagV6P, but not FlagV1P, was able to bind to *C. jejuni* strain 11168. These filaments were confirmed to be flagella with a polyclonal antibody specific to 81–176 flagella (Figure 5C). Western blotting of whole cell lysates of *C. jejuni* 81–176 and *C. jejuni* 11168 with FlagV6P and FlagV1P demonstrated a similar pattern of reactivity with denatured flagellin monomers (data not shown). As with binding to flagella filaments on the cell surface, only FlagV6P was able to bind to the flagellin monomers of both *C. jejuni* strains 81–176 and 11168 by Western blotting. In contrast, FlagV1M was only able to detect flagellin monomers from *C. jejuni* 81–176.

### Cross-Reactivity of Anti-Flagella Pentabodies with *Campylobacter* Strains

**Figure 6:**
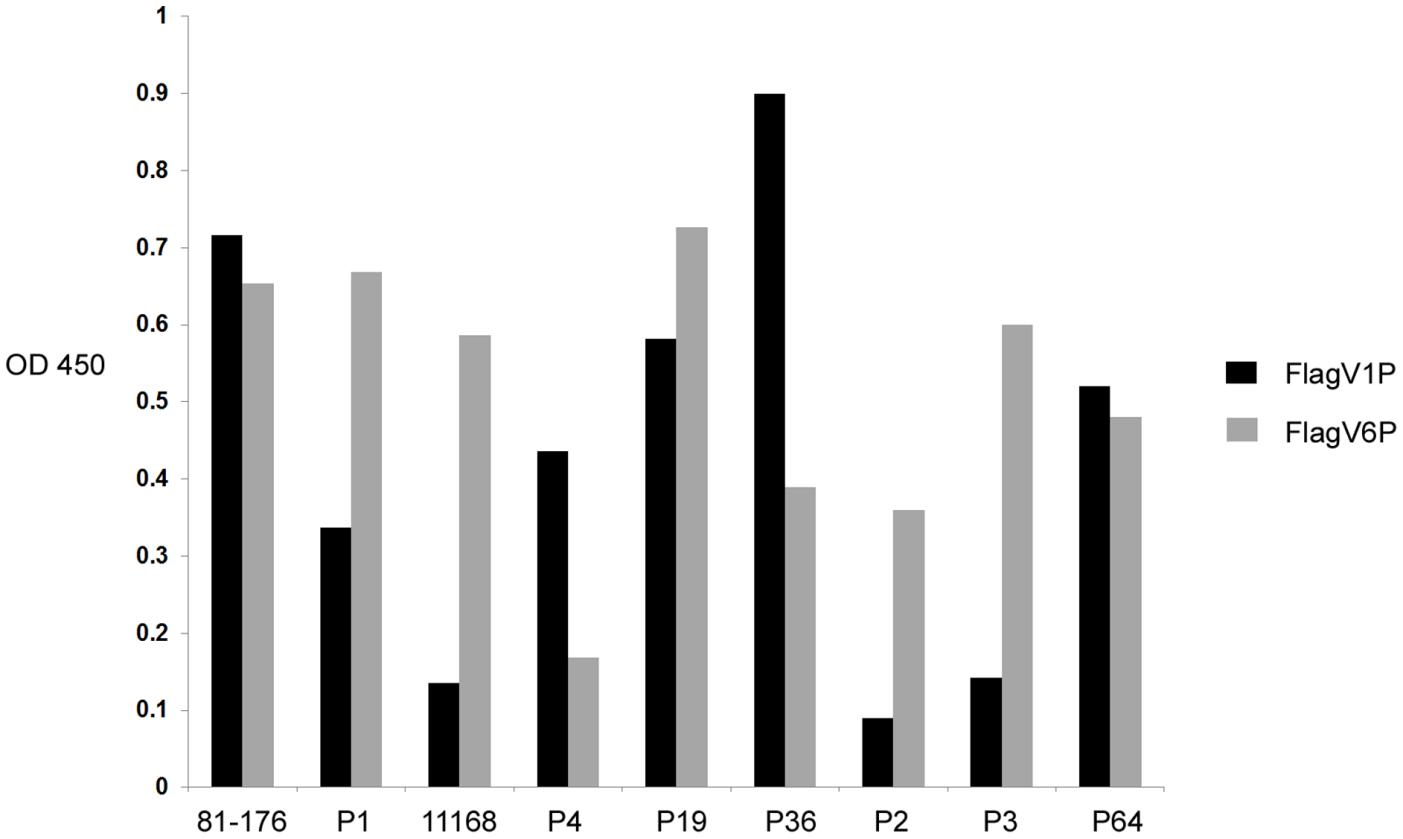
ELISA demonstrating binding of Flag1V1P and FlagV6P to flagella isolates from 9 different strains of *C. jejuni*. ELISA was performed according to the standard procedure. In brief, wells were coated with 10 µg of *C. jejuni* flagella proteins and binding was detected using either FlagV1P or FlagV6P pentabodies. Absorbance values indicate an average of two independent experiments.

The flagella-specific pentabodies, FlagV1P and FlagV6P, were tested in ELISA with purified flagella prepared from 9 *C. jejuni* strains. All strains used in the assay were human isolates except strain P2 which was a calf isolate. The bacterial strains tested were 81–176: ATCC BAA-2151, 11168: ATCC 700819, and Penner serotype strains [60]: P1: ATCC 43429, P2: ATCC 43430, P3: ATCC 43431, P4: ATCC 43432, P19: ATCC 43446, P36: ATCC 43456, and P64. ELISA demonstrated a distinct pattern of binding of FlagV1P and FlagV6P to flagella from different strains (Figure 6). FlagV1P interacts strongly with 81–176 (the strain used for immunization) and five other strains, but it does not bind to strains 11168, P2 and P3 under the conditions tested. FlagV6P reacted with all strains tested except strain P4.

### Anti Flagellin Pentabodies Disrupt *Campylobacter* Motility

**Figure 7:**
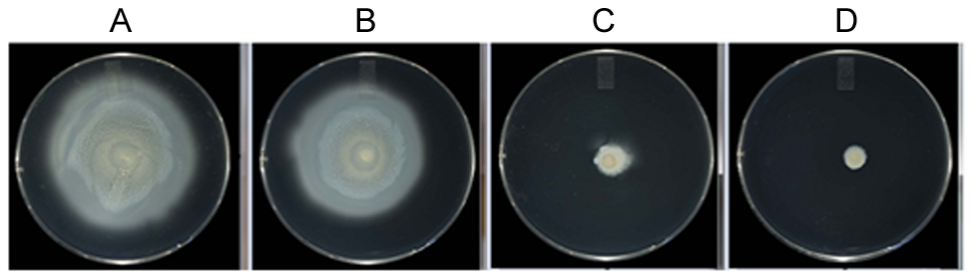
Motility assay of *C. jejuni* strain 81–176 in the absence or presence of pentabodies. *C. jejuni* 81–176 was incubated for 30 min with (A) buffer, (B) control unrelated pentabody at 0.5 µg/µl, (C) FlagV1P at 0.25 µg/µl, and (D) FlagV1P at 0.5 µg/µl. Images were taken after 53 h of incubation.

V_H_H and pentabody-mediated inhibition of *Campylobacter* motility was studied using a standard soft agar plate assay. Co-incubation of *C. jejuni* 81–176 with FlagV1M, FlagV1P and FlagV6P monomers or pentabodies (Figure 7, Table 2) showed a marked reduction in bacterial motility. *C. jejuni* strain 11168 was also examined and demonstrated a similar inhibition in motility on the plate assay with only the FlagV6P antibody (data not shown). The impact of FlagV1M and FlagV1P on disrupting the motility seemed to last for up to 53 h post incubation.

**Table 2:** *C. jejuni* 81–176 motility on plates after incubation with FlagV1 and FlagV6 antibodies.

Treatment	Diameter (mm) ±SD - 24 h	Diameter (mm) ±SD - 48 h
PBS	26.6±2.25	82±3.3
Unrelated pentabody	24±2.7	67.3±4.5
FlagV1M	8.6±1.25*	19.5±0.5*
FlagV1P	8.8±0.76*	45.16±5*
FlagV6P	8.75±0.35*	45.5±14.08
FlagV1P + FlagV6P	9±1.32*	28±3.04*

The diameter of the circles representing the spread of bacteria from the inoculum site was measured.

Asterisk indicates statistical significance of Flag antibody treatments vs the control unrelated pentabody.

The activity of the anti-flagellin pentabodies was also tested on *Campylobacter coli* strain VC167. Only FlagV6P was effective in reducing motility (Table 3). No significant effect on motility was observed when *Salmonella enterica* serovar Typhimurium was incubated with the anti-flagellin pentabodies (data not shown).

**Table 3:** Motility assays show the cross-reactivity of the pentabodies FlagV1P and FlagV6P with *C. coli VC167*.

Treatment	diameter (mm) ±SD- 24 h	diameter (mm) ±SD- 48 h	diameter (mm) ±SD- 72 h
PBS	12.5±0.5	25.3±1.52	43.6±4.5
FlagV1P	10.6±0.6*	22±3	42.3±4.1
FlagV6P	5.6±0.66**	11.5±1.32**	25.6±3.05**

Significant reduction in motility of *C. coli* was noticed with FlagV6P pentabody. The values were subjected to the Student's *t*-test for statistical analysis.

*p value <0.05.

**p value <0.005.

### Anti-Flagella Pentabody Reduce *Campylobacter* Colonization of the Chicken Gut

**Figure 8:**
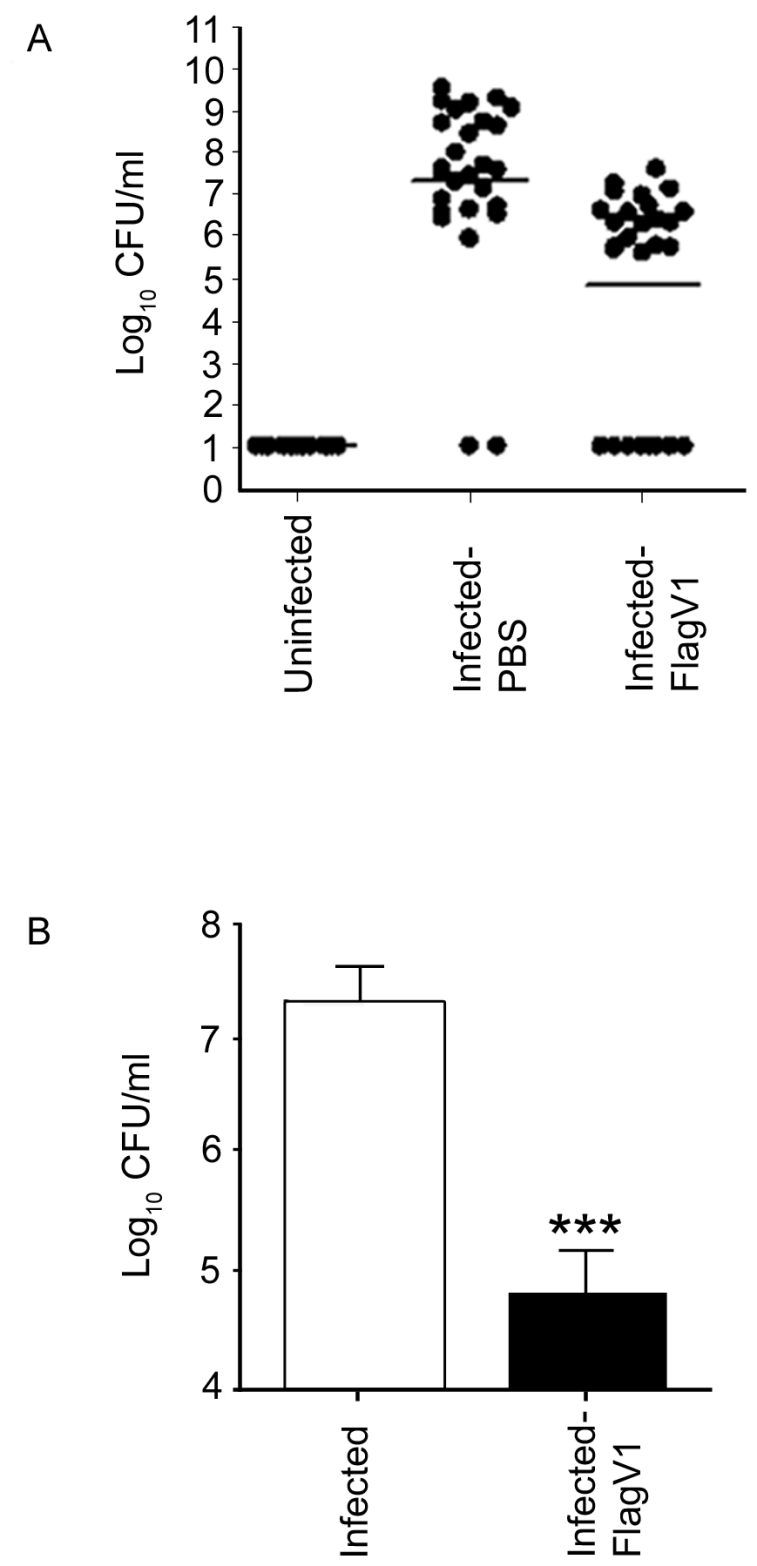
Effect of oral administration of FlagV1P on the levels of *C. jejuni* colonization of chickens inoculated with 10^8^ *C*. *jejuni* cells. At 1*C. jejuni*, chickens (n = 28/group, 14 chickens in each of 2 containment units) received 300 µl FlagV1P. An uninfected, negative control group was also included (n = 15). (A) Bacterial burdens (mean ±SEM) in the individual ceca are indicated by scatterplot and the mean cecal bacterial burden is denoted by a horizontal line. (B) Bacterial colonization levels (mean ±SEM) in the ceca of chickens treated with PBS (open bars), FlagV1P (closed bars). *** p<0.001, one-way ANOVA, followed by Bonferroni multiple comparison test. The detection limit of *C. jejuni* in the cecum sample was determined to be 10 CFU/ml.

The efficacy of the pentabodies in reducing *C. jejuni* colonization was evaluated in 2-day-old SPF chickens (Figure 8). Oral administration of FlagV1P at 1 h, 24 h and 48 h after inoculation of chickens with 10^8^ CFU of *C. jejuni* significantly reduced bacterial loads in the ceca (Figure 8, p<0.001). A negative control group was also included in each experiment which showed baseline levels of *C. jejuni* in the ceca of uninfected chickens.

### Administration of Anti-Flagella Pentabody Does not Impact Chicken Weight Gain

**Figure 9:**
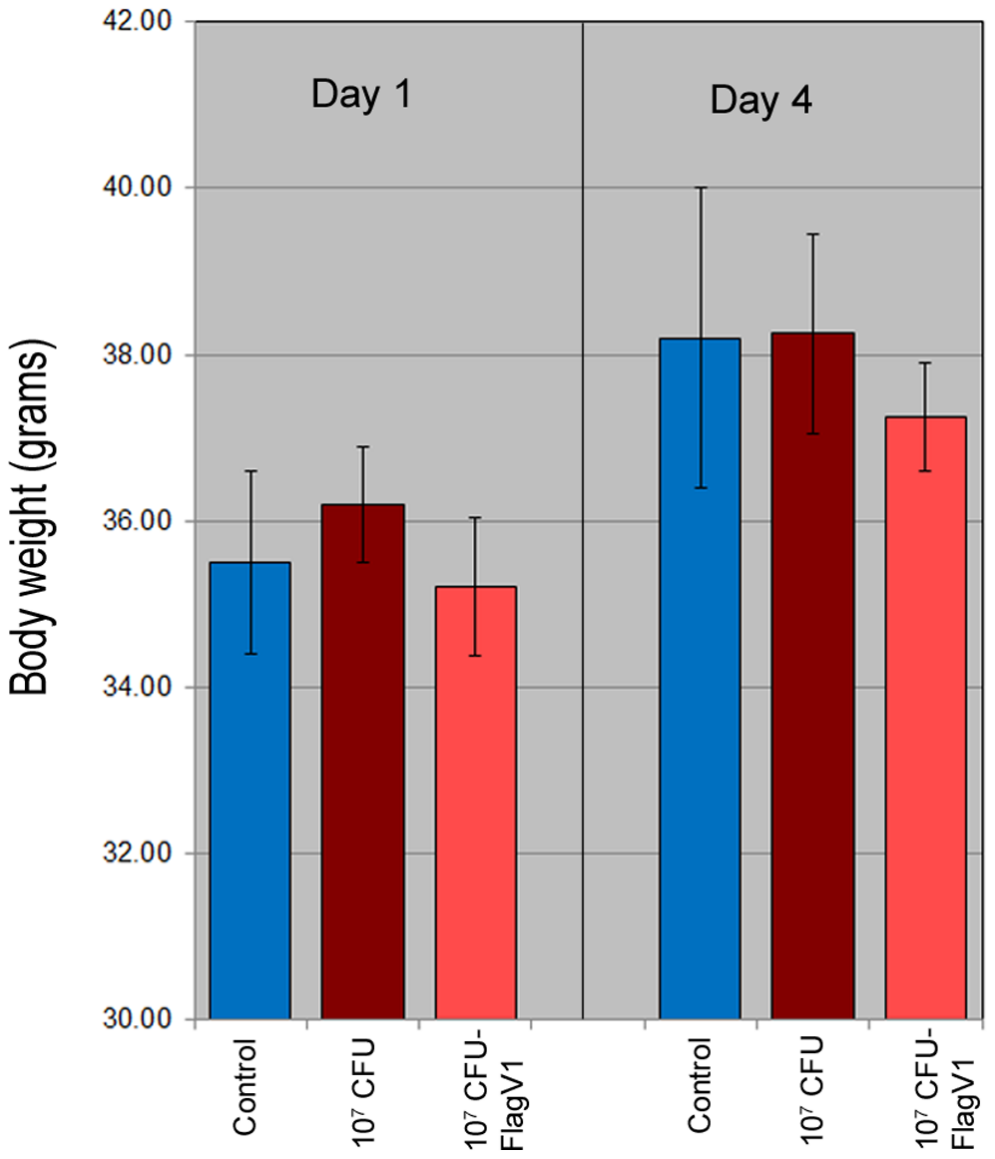
The effect of administration of flagella-specific antibodies on chicken body weight. Chicken were weighed one day and four days after challenging with *C. jejuni* alone or *C. jejuni* followed by pentabody administration. PBS was used as a control and body weights (in grams) were measured at day one and day four. The average body weight ± standard deviation of the values obtained from 28 replicates is shown for each group. No significant differences were found between the groups.

The effect of gavaged FlagV1 pentabody on chicken body weight was investigated by measuring body weights at one day and four days after challenging with *Campylobacter* alone or when the pentabody was administered orally. As a control, saline buffer was used and the average body weights were measured (Figure 9). No significant differences were found between the groups.

### Orally-Administered Flagella-Specific Pentabody Localize to the Chicken Ileum and Cecum

**Figure 10:**
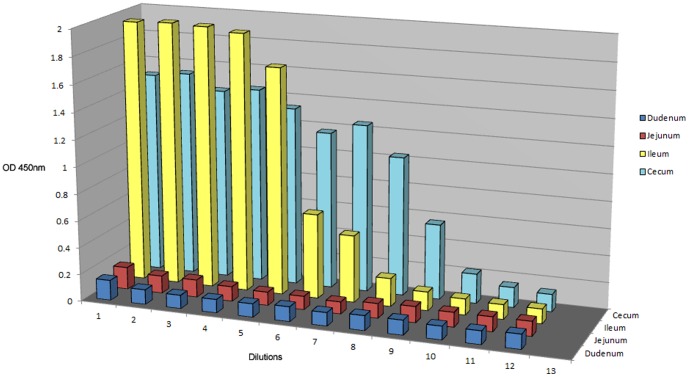
Detection of anti-*C. jejuni* pentabodies (FlagV1P) in different sections of the chicken intestinal tract. Separate chickens were gavaged with the flagella-specific pentabody according to the schedule that was followed in our chicken colonization studies. Intestinal fluids were collected from the cecum, ileum, jejunum and duodenum and used in 2-fold serial dilutions {1/2 (no. 2)-1/2048 (no. 12)} in ELISA using anti-verotoxin antibody to capture the pentabodies and anti-His-HRP antibody conjugates for their detection. Chickens were gavaged with 1 mg of FlagV1P.

The chicken intestinal tract was dissected to examine for the presence of gavaged pentabodies (FlagV1P). Intestinal fluids were collected from the cecum, ileum, jejunum and duodenum and used in 2-fold serial dilutions in a sandwich ELISA where anti-verotoxin antibody was used as the capturing antibody and anti-His-HRP antibody for detection (Figure 10). On average, higher concentrations of FlagV1P pentabody were found in the Ileum and cecum fluid extracts compared to the other regions of the intestine. Intestinal fluids from control chicken showed no significant binding in the same ELISA.

## Discussion

Single-domain V_H_H antibodies are emerging as novel biological reagents against bacterial and viral infections [61], [62]. Their small size facilitates binding to epitopes that are inaccessible to conventional antibodies and their unique physical properties such as resistance to proteolysis, denaturation and aggregation allows potential applications in oral delivery of therapeutics in humans or livestock. In addition, they can be economically produced in large amounts in bacteria and yeast for various applications.

In the present study we used phage display technology to construct and screen an immune library of V_H_H antibodies against *C. jejuni* flagella. We demonstrated that V_H_Hs and their pentameric versions were able to bind to flagella and were effective in significantly lowering *C. jejuni* colonization levels in chickens when administered orally. The rationale for our experimental design is based on a number of previous studies that demonstrated a direct correlation between resistance to or delay in colonization by *C. jejuni* and the presence of the organism or flagella-specific antibodies in the serum/intestinal secretions in both animal models and in humans [19], [47]–[51], [65]. We believe that the unique features of *Camelidae* single domain antibodies, in particular, their robust neutralization power and low production cost, may provide excellent opportunities for antibody-based reduction of *Campylobacter* colonization.

The isolated V_H_Hs were well expressed in *E. coli*, up to 10–80 mg/litre in shaker flask culture, and size exclusion chromatography demonstrated that all exist as non-aggregating monomers (results not shown). The antibodies also reacted strongly with the protein target with affinities in the range of 20–30 nM. Deduced amino acid sequences of the antibodies showed no significant sequence homology between the CDRs of FlagV1 and FlagV6. This was additionally supported by our SPR data indicating binding of the two V_H_Hs to different epitopes. Analyses of the V_H_H sequences suggests that the FlagV1M V_H_H belong to subfamily I, whereas the FlagV6M V_H_H contains most of the subfamily II-specific residues based on the V_H_H classification system described by Harmsen *et al.*, 2000 [63]. The anti-flagella V_H_Hs isolated here are among the highest affinity single-domain antibodies characterized to date.

*In vitro* motility studies showed that FlagV1P and FlagV6P recognized native flagella and upon binding prevented cell motility of *C. jejuni* 81–176. The interference with bacterial motility was even more pronounced with FlagV1M compared to FlagV1P 53 h after incubation. This might be explained by potentially higher stability of the FlagV1M V_H_H on the assay plate. The antibodies were also able to recognize other strains of *C. jejuni*; and FlagV6P was even able to reduce the motility of *C. coli* strain VC167. Variability in the pattern of binding to different strains of *C. jejuni* was also identified when flagella from 9 different strains were tested by ELISA, suggesting that FlagV1 and FlagV6 CDRs target different epitopes on the flagella. This was further confirmed by SPR analyses which indicated that distinct epitopes were targeted by FlagV1M and FlagV6M. We are currently investigating the actual peptide and/or carbohydrate epitopes responsible for pentabody binding.

As shown by fluorescence microscopy, FlagV1P and FlagV6P bind to the flagella at the poles of *C. jejuni*. As with the ELISA, FlagV1P was able to bind 81–176, but not 11168, whereas FlagV6P was able to bind both strains. As this binding was lost with a non-flagellated mutant (*flaA^−^, flaB^−^*), it confirmed the binding was specific to flagellin proteins.

To determine the efficacy of the anti-flagella pentabody in reducing *C. jejuni* colonization, chicken studies were performed using FlagV1P due to the slower off-rate of FlagV1 compared to FlagV6 (Figure 3). The antibodies were used in the chicken studies with the assumption that the bacterial agglutination or motility inhibition caused by the V_H_H domains of the pentabodies would impair the ability of *C. jejuni* to colonize the chicken gastrointestinal tract. Flagellin is considered a major *C. jejuni* virulence factor and motility is important for maneuvering through the viscous intestinal mucus [64], [65]. Our experiments demonstrated an approximately 3 log reduction in the rate of colonization in the group treated with FlagV1P. This further substantiates the important role of flagella in colonization of *C. jejuni*. Our antibody localization studies using ELISA suggested that at least a percentage of the pentabodies remain functional after the passage through the GI tract and reach the cecum, which is the principal site of *C. jejuni* colonization [24], [66]. The demonstration that the flagellar pentabodies also inhibit motility could further contribute to their increased efficacy in reducing *C. jejuni* colonization in chickens.

While this is, to our knowledge, the first demonstration of the efficacy of V_H_H-based molecules in the reduction of *C. jejuni* levels in poultry, there are other reports of their successful application in reducing pathogen levels at mucosal surfaces. Harmsen *et al*. (2005) [67] observed that V_H_Hs specific for the F4 fimbriae of enterotoxigenic *E. coli* (ETEC) were highly effective in reducing pathogen binding to piglet intestinal brush borders *ex vivo* and somewhat effective in reducing ETEC-induced fluid loss when perfused into a piglet jejunal segment. The research team later reported an improvement of the *in vivo* function of the V_H_Hs by isolating protease-resistant versions of V_H_Hs specific to F4 fimbriae of ETEC [68]. Preliminary studies have indicated that the pentabodies used in the current work are susceptible to degradation by trypsin, chymotrypsin and pepsin (data not shown) yet still demonstrated efficacy in *in vivo* studies. We expect a second-generation protease resistant version of the V_H_Hs and their pentabodies used in this study will be even more effective in reducing the colonization of *C. jejuni* in the chicken cecum, and, therefore, oral application of V_H_Hs and pentabodies might be considered as novel strategies for reducing *C. jejuni* contamination of poultry products. Future experiments will be designed to address the feasibility of obtaining protease-resistant pentabodies and evaluating their effectiveness in the prevention of *C. jejuni* colonization over the full growth cycle of the chicken, as well as in treatment of chickens that have already been colonized with the bacteria.

## Materials and Methods

### Ethics Statement

The animals were maintained and used in accordance with the recommendations of the Canadian Council on Animal Care Guide to the Care and Use of Experimental Animals. The experimental procedures were approved by the institutional animal care committee.

### Antigen Preparation and Immunization

*C. jejuni* 81–176 flagella were isolated as described previously [69]. Briefly, bacteria were cultured 16 h in Mueller-Hinton broth (Sigma-Aldrich, St. Louis, MO, USA) under microaerobic conditions. Approximately 5×10^12^ cells were then harvested by centrifugation (11,000×g, 30 min) and resuspended in 100 ml of Tris-buffered saline solution. Flagella were sheared using a Waring blender on ice. Cell debris was pelleted by two low speed centrifugations (10,000×g, 20 min) and the supernatant was transferred to an ultracentrifugation tube. Flagella were pelleted by centrifugation for 1 h at 100,000×g. Further purification was done by resuspending the pellet in 1% (vol/vol) SDS in distilled water and re-pelleting the flagella by centrifugation. Pellets were finally resuspended in 200–500 µl of dH_2_O.

A male llama (*Lama glama*) was immunized subcutaneously with *C. jejuni* flagella. Seven injections were performed consisting of 100 µg of the antigen in a total volume of 0.5 ml mixed with an equal volume of either complete (day 1) or incomplete (days: 21, 35, 49, and 63) Freund's adjuvant (Sigma-Aldrich). The last two injections (days: 76 and 90) were performed without adjuvant. Pre-immune blood (15–20 ml) was collected before the first injection with subsequent collections on days 21, 56, 70 and 95. The specific immune response to flagella was analyzed by ELISA using total pre-immune and immune sera as described below. Serum from day 95 was fractionated as described before [54]. Protein G and A columns (GE Healthcare, Pittsburgh, PA, USA) were used for serum fractionation according to the manufacturer's instructions and separated fractions were adjusted to pH 6 with 1 M Tris/HCl, pH 8.8, and dialyzed against PBS at 4°C overnight. Individual heavy fractions (G1, A1 and A2) and G2 (conventional IgG) were analyzed for specific binding to flagella antigen by ELISA. Briefly, 96-well Maxisorp™ plates (Nalgene Nunc International, Rochester, NY) were coated overnight at 4°C with 5 µg/ml of flagella antigen in PBS. Wells were rinsed and blocked with 200 µl of 1% casein. Different dilutions of purified IgG fractions (G1, G2, A1 and A2) were added and incubated at room temperature for 1.5 h. Wells were washed with PBS with 0.05% Tween-20, and incubated with goat anti-llama IgG (H+L) (1∶1,000 in PBS) (Bethyl Laboratories, Montgomery, TX) followed by swine-anti-goat-HRP (1∶3,000 in PBS) (Cedarlane, Burlington, ON, Canada). Binding was detected by adding 100 µl Tetramethylbenzidine (TMB) peroxidase substrate per well (Kirkegaard and Perry Laboratories, Gaithersburg, MD, USA). Reactions were stopped by adding 100 µl 1 M phosphoric acid and A_450_ was measured using a Bio-Rad ELISA plate reader.

### Phage Library Construction and Panning

A phage display library was constructed as previously described [70]. In brief, total RNA was isolated from approximately 2×10^7^ lymphocytes collected on day 95 post-immunization start using a QIAamp RNA blood mini kit (Qiagen, Mississauga, Ontario, Canada). First-strand cDNA was synthesized with oligo(dT) primer using 5 µg total RNA as template according to the manufacturer's recommendations (GE Healthcare). Immunoglobulin variable domains and part of the constant region (VH/V_H_H-CH2) DNA were amplified by PCR using oligonucleotides MJ1-3 (sense) and two CH2 domain antisense primers CH2 and CH2b3 as previously described [70]. Briefly, the PCR reaction mixture was set up in a total volume of 50 µl with the following components: 1–3 µl cDNA, 10 pmol of MJ1-3 primer mixture, 10 pmol of either CH2 or CH2b3 primers, 5 µl of 10× reaction buffer, 1 µl of 10 mM dNTP, 2.5 units of Taq DNA polymerase (Hoffmann-La Roche Limited, Mississauga, ON, Canada). The PCR protocol consisted of an (i) initial step at 94°C for 3 min, followed by (ii) 30 cycles of 94°C for 1 min, 55°C for 30 s, 72°C for 30 s and (iii) a final extension step at 72°C for 7 min. The heavy chain fragments (550–650 bp in length) were gel-purified using a QIA quick gel extraction kit (Qiagen). The variable regions of the heavy chain antibodies (IgG2 and IgG3) were re-amplified in a second PCR reaction using MJ7and MJ8 oligonucleotides and the same conditions as described above [70]. The amplified PCR products were purified with a QIAquick PCR purification kit (Qiagene), digested with *Sfi*I (New England Biolabs, Pickering, Ontario, Canada) and re-purified using the same kit. Twelve micrograms of digested V_H_H fragments were ligated with 40 µg (3∶1 molar ratio, respectively) *Sfi*I-digested pMED1 phagemid vector [70] using LigaFast Rapid DNA ligation system (Promega, Madison, WI). Electrocompetent TG1 *E. coli* cells (Stratagene, La Jolla, CA) were transformed with using the ligated products as described previously and a library of approximately 5×10^7^ transformants was obtained. The V_H_H fragments from 40 colonies were PCR-amplified and sequenced to analyze the complexity of the library. The library was expanded by culturing for 3–4 h in 2× YT (yeast extract tryptone) [71] containing ampicillin (100 µg/ml)/glucose (2% w/v) medium at 37°C. The bacterial cells were pelleted, resuspended in the same medium with 20% glycerol and stored at −80°C.

Panning was performed for a total of four rounds against the flagella as essentially described by Arbabi Ghahroudi et al. 2009 [70]. In summary, 1 ml of the library stock (5×10^10^ bacterial cells) was grown for 1–2 h at 37°C, with shaking at 250 rpm in 2× YT/Amp-Glucose (2% w/v) medium (A_600_ = 0.4–0.5) and infected with M13KO7 helper phage (20∶1 phage to cells ratio) (New England Biolabs) for 1 h at 37°C. After centrifugation of the culture at 4°C, the infected cell pellets were resuspended in 200 ml of 2× YT/Amp with 50 µg/ml kanamycin and incubated for 16 h at 37°C with shaking at 250 rpm. The phage particles in the culture supernatant were precipitated with polyethylene glycol and the phage pellets were resuspended in 2 ml of sterile PBS and the phage titre was determined as previously described [70]. For panning, 96-well Maxisorp™ plates were coated with flagella antigen and the wells were rinsed with PBS and blocked with PBS/1% (w/v) casein for 2 h at 37°C. Approximately 10^12^ rescued phage particles were added to the blocked wells and incubated for 2 h at 37°C. The wells were washed 5× with PBS with 0.1% Tween-20 and 5× with PBS. The bound phages were eluted with 0.1 M triethylamine for 10 min, neutralized with 1 M Tris-HCl, pH 7.4 and used to infect exponentially growing *E. coli* TG1 cells. After 30 min incubation at 37°C, the cells were superinfected with M13KO7 for an additional 15 min and grown in 2× YT containing ampicillin (100 µg/ml) and kanamycin (50 µg/ml) overnight at 37°C. Panning was continued for three more rounds following the same procedures except that the antigen concentration was reduced to 20, 15, and 10 µg/well and the washing cycle was increased to 7, 10 and 12× with PBS-T and PBS for the second, third and fourth rounds of panning, respectively. After four rounds of panning, 48 colonies were randomly picked and phage were prepared and tested for binding to flagella using a published phage ELISA protocol [70]. All positive clones were sequenced and unique sequences for each antigen were selected for sub-cloning and large-scale expression and purification. Two anti-flagella V_H_Hs were isolated and further characterized. The sequencing data were deposited in GenBank™, accession numbers KF812523 (FlagV1M) and KF812524 (FlagV6M).

### Expression and Purification of Soluble and Pentameric V_H_Hs

V_H_H DNA was PCR amplified from the pMED1 phagemid vector using oligonucletides *Bbs*I-V_H_H (5′-TATGAAGACACCAGGCCCAGGTAAAGCTGGAGGAGTCT- 3′) and *Bam*HI-V_H_H (5′-TTGTTCGGATCCTGAGGAGACGGTGACCTG-3′) (for the monomer) or *Apa*I-V_H_H (5′-ATTATTATGGGCCCTGAGGAGACGGTGACCTGGGTC-3′) as the reverse primer with *Bbs*I (for the pentamer). These PCR fragments were digested with *Bbs*I-*Bam*HI or *Bbs*I-*Apa*I restriction enzyme pairs (New England Biolabs) and ligated separately with either a pUC derivative pSJF2H (monomer) or pVT2 (pentamer) expression vectors [70]. Upon ligation, all plasmids were used to transform electrocompetent TG1 *E. coli* followed by selection on LB ampicillin agar plates. Colonies were screened by PCR for inserts and the DNA was sequenced.

V_H_H antibodies were expressed in bacteria and purified as previously described [70]. In brief, protein expression was induced by addition of IPTG to the media, and the periplasmic contents were extracted from the cell pellet. Briefly, the cell pellets of monomeric V_H_H cultures were resuspended in 20 ml of ice cold TES (0.2 M Tris-HCl pH 8.0, 20% (w/v) sucrose, 0.5 mM EDTA) and incubated on ice for 30 min. Next, 30 ml of ice-cold 1/8 TES (diluted in dH_2_O) was added followed by incubation for 30 min on ice, and then centrifugation at 14,000×g for 30 min at 4°C. The resulting supernatant containing V_H_Hs was dialysed overnight against the metal-affinity chromatography (IMAC) buffer A (10 mM HEPES pH 7.0, 500 mM NaCl) prior to loading on the HiTrap™ Chelating HP columns (GE Healthcare) for V_H_H purification as described previously [70]. Eluted protein fractions were analyzed by SDS-PAGE and Western blotting before being pooled and dialysed against PBS. Briefly, protein samples were run on a 12.5% acrylamide gel in duplicate and one stained with Coomassie blue dye. The protein on the second gel was transferred onto a nitrocellulose membrane using a Trans-BlotTM SD for semi-dry Western blotting (Bio-Rad Laboratories). The membrane was incubated with mouse anti-6× His monoclonal antibody followed by goat-anti-mouse alkaline phosphatase (AP) conjugate and developed by addition of AP substrate buffer as previously described [70]. For pentabody isolation, the cells were lysed using a lysozyme lysis method and the cell lysates were centrifuged and filtered through 0.22 µm membrane filters prior to antibody purification by IMAC as described previously [70].

### ELISA

ELISA was performed to determine specific binding of the monomer and pentamer V_H_Hs to the protein target as described above except that after washing the plate with PBST and blocking with PBS-casein (1%), a 5 µg/ml solution of either anti-flagella monomer (FlagV1M) or the corresponding pentamer (FlagV1P) was added to the respective wells and incubated for 1 h at 37°C. Wells were washed with PBST (0.05% v/v Tween-20) and rabbit anti-His_6_ antibody conjugated to HRP (1∶5000 in PBS) (Bethyl Laboratories) was added followed by incubation for 1 h at room temperature. Binding was detected with the TMB substrate (Kirkegaard and Perry Laboratories), the reaction was stopped with 1 M H_3_PO_4_, and A_450_ was measured using an ELISA plate reader as described above.

To determine the cross-reactivity of the purified anti-flagella pentamers (FlagV1P or FlagV6P) with different strains of *C. jejuni*, flagella were prepared from different strains and used for coating of the microtitre plates. ELISA was performed as described above. The Campylobacter strains were originally obtained from ATCC and kindly provided to us by Dr. Michel Gilbert, NRC, Canada: 81–176: ATCC BAA-2151, 11168: ATCC 700819, and Penner serotype strains [60]: P1: ATCC 43429, P2: ATCC 43430, P3: ATCC 43431, P4: ATCC 43432, P19: ATCC 43446, P36: ATCC 43456, and P64: originally from Erasmus University, also a gift from Dr. Michel Gilbert.

### Surface Plasmon Resonance

For surface plasmon resonance, monomeric and pentameric V_H_Hs were passed through size exclusion columns, Superdex 75 and 200 (GE Healthcare), respectively, in 10 mM HEPES, pH 7.4, containing 150 mM NaCl and 3 mM EDTA to remove aggregates. Monomeric and pentameric V_H_H peak fractions were collected and protein concentrations determined from A_280_ measurements. Purified flagella protein was biotinylated by reaction with Pierce EZ-Link Sulfo-NHS-LC-LC-biotin (GE Healthcare) followed by dialysis against PBS to remove excess unincorporated biotin. SPR analyses were performed using a Biacore 3000 instrument (GE Healthcare). All measurements were conducted at 25°C in 10 mM HEPES, pH 7.4, containing 150 mM NaCl, 3 mM EDTA and 0.005% surfactant P20 (GE Healthcare). Approximately 700–900 RUs of biotinylated flagella was captured on an SA sensor chip (GE Healthcare) at a flow rate of 5 µl/min. Various concentrations of the antibodies were injected over flagella-SA surface at a flow rate of 40 µl/min. Surfaces were regenerated by washing with running buffer and data were analyzed with BIAevaluation 4.1 software. Epitope mapping of flagella-specific antibodies was performed using Biacore co-injection experiments. Briefly, 60 µl of the anti-flagella V_H_Hs diluted in HBS-EP buffer to a concentration of 50× their respective K_D_ values were injected over 700 RUs of immobilized biotinylated flagella at 30 µl/min. A FlagV1 injection was followed by a FlagV6 injection.

### Western Blotting

Whole cell lysates of overnight cultures of *C. jejuni* strains 81–176 and 11168, *C. coli* strain VC167 and the flagellar mutant strain 81–176 *flaA^−^/flaB^−^* were separated on 12.5% SDS polyacrylamide gels under reducing conditions and proteins transferred to nitrocellulose membranes. Membranes were blocked with 3% (w/v) bovine serum albumin (BSA) in phosphate buffered saline (PBS) and hybridized with FlagV1P and FlagV6P pentabodies for 1 h at room temperature. After washing five times with PBST, membranes were incubated with anti-His AP conjugates (diluted 1∶5,000 in blocking buffer) (Abcam, Cambridge, MA). Finally, the membranes were washed four times and incubated with AP substrate (Bio-Rad Laboratories, Mississauga, Canada) for 10 min. The AP reaction was stopped by rinsing the membranes with distilled H_2_O and air dried.

### FITC Labelling of Pentabodies and Fluorescence Microscopy

FlagV1P and FlagV6P were labelled at a concentration of 2 mg/ml with FITC using the FITC-labeling kit from Invitrogen according the manufacturer's instructions. The labeled pentabodies were dialysed against PBS several times to remove unincorporated FITC. *C. jejuni* 81–176 wild type; 81–176 *flaA^−^flaB^−^* and *C. jejuni* 11168 were cultured in Mueller-Hinton broth (Sigma-Aldrich) until reaching exponential growth phase, pelleted and fixed in 3% formalin overnight. The cells were washed with PBS to remove formalin, and then 10 µl of cells at a final concentration of ∼1×10^8^/ml were air dried on glass coverslips. Non-specific binding was blocked with 50 µl 5% Bacto skim milk (Difco)-PBS for 1 h at room temperature. The cells were incubated for 1 h at room temperature in 50 µl FITC labelled FlagV1P or FlagV6 pentabody diluted in PBS to 80 µg/ml. The cells were washed with PBS/0.1% Tween and then mounted onto glass slides with addition of Vectashield-DAPI (Vector Laboratories, Burlington, Canada) mounting medium. The slides were examined with a Zeiss Axiovert 200 M microscope (Zeiss, Toronto, Canada). The experiment was done in duplicate, on at least three independent occasions, with at least three fields of view on each coverslip imaged. To confirm the pattern of fluorescence observed were flagella filaments; a control of a rabbit polyclonal anti-81-176 flagellin was used at 1∶1,000 dilution in PBS, in place of the FlagV1 and FlagV6P pentabodies. The cells were washed with PBS/0.1% Tween, then incubated for 45 min at room temperature with 50 µl Alexafluor 488/FITC goat anti-rabbit IgG (Invitrogen) diluted 1∶1,000 in PBS. The slides were washed with PBS/0.1% Tween then mounted as stated above.

### Motility Assay

A motility assay was performed as described previously [72]. Antibodies, at a final concentration of 1 µg/µl, were incubated with *C. jejuni* or *C. coli* (5×10^7^ CFUs) at RT for 30 min. The mixtures were plated in the center of a petri dish containing Mueller-Hinton agar (0.4%) and incubated at 37°C under microaerobic conditions (5% O_2_, 10% CO_2_, and 85% N_2_). Bacterial motility was determined by measuring the diameter of the growth at 24 h, 48 h, and 72 h after inoculating the bacteria.

### *C. jejuni* Colonization of Specific Pathogen-Free Leghorn Chicks

Cultures for chick colonization experiments were prepared by harvesting *C. jejuni* 81–176 grown for 18 h in Mueller Hinton broth (Sigma). Bacterial cells were diluted in PBS and maintained on ice until immediately before use. One day old SPF leghorns (mixed sex) were obtained from the hatchery at the Canadian Food Inspection Agency (CFIA, Ottawa, ON, Canada). The chicks were randomly assigned into negative control, positive control, and treatment groups, weighed, ID tagged, housed in animal containment units and provided with feed and water *ad libitum*. The units were housed in an environmentally controlled level 2 bio-containment room. On arrival, 10% of the birds were randomly tested for colonization with *C. jejuni*. On day two, positive control and treatment groups were orally challenged with 300 µl *C. jejuni* 81–176 (10^8^ cfu/ml). Positive control groups received 300 µl PBS and treatment groups received 300 µl (1 mg) of the anti-flagella pentabody at 1 h, 24 h, and 48 h after the challenge. Birds were euthanized 48 h after pentabody treatments by cervical dislocation according to the approved guidelines of the Canadian Council for Animal Care. Ceca were aseptically collected for quantitative assessment of colonization. Cecal contents were serially plated onto Karmali agar (Oxoid) and *C. jejuni* counts were done after incubation for 2 days at 37°C under microaerobic conditions. The chicken body weights (in grams) were also measured on day 1 and 4 after challenging with *C. jejuni* alone or following pentabody administration, to determine the impact of the experimental treatments.

### Localization of Pentabodies in the Chicken GI Tact by Sandwich ELISA

The presence and approximate concentration of the pentabodies in different regions of the chicken GI tract were evaluated by ELISA assay. Wells of Maxisorp™ ELISA plates were coated with 1 µg of mouse monoclonal anti-verotoxin antibody recognizing the verotoxin component of FlagV1P and FlagV6P, overnight at 4°C. After blocking with PBS-casein (1%), intestinal fluids collected from the cecum, ileum, jejunum and duodenum regions were added to the ELISA plate wells in 2-fold serial dilutions (1/2-1/2048) and incubated at 37°C for 1 h. Subsequently, the pentabodies were detected using rabbit anti-His_6_ IgG conjugated to HRP (1∶5,000 in PBS) (Bethyl Laboratories) and TMB substrate. The reactions were stopped and absorbance measurements were made at 450 nm.

### Statistical Analyses

Data are presented as means ±SEM for each chicken group, unless otherwise specified. Differences in tissue bacterial burdens were assessed by the Student's *t*-test or one-way analysis of variance (ANOVA) followed by Bonferroni's *post-hoc* multiple comparison tests, when appropriate. Differences were considered significant when p<0.05.
